# Robotic total gastrectomy with *π*-shaped esophagojejunostomy using a linear stapler as a novel technique

**DOI:** 10.1186/s12957-018-1542-z

**Published:** 2018-12-21

**Authors:** Shangxin Zhang, Junaid Khaliq, Deguan Li, Xingwang Jiang, Ruochuan Sun, Yongxiang Li

**Affiliations:** Department of Gastrointestinal Surgery, Department of General Surgery, First Affiliated Hospital of Anhui Medical University, 218 JiXi Avenue, Hefei, 230022 Anhui China

**Keywords:** *π*-Shaped esophagojejunostomy, Robotic total gastrectomy, Intracorporeal esophageojejunostomy

## Abstract

**Objective:**

To evaluate the intraoperative and short-term postoperative outcomes of a novel robotic intracorporeal *π*-shaped esophagojejunostomy (EJS) after D2 total gastrectomy (TG) using the Da Vinci robotic surgical system for intracorporeal anastomosis after TG.

**Background:**

Intracorporeal *π*-shaped EJS, using a linear stapler, was recently reported for laparoscopic total gastrectomy in patients with gastric cancer. However, robotic intracorporeal *π*-shaped EJS using a linear stapler has not been reported. This report aimed to describe the use of a novel technique for *π*-shaped EJS using the Da Vinci robotic system.

**Methods:**

Robotic intracorporeal *π*-shaped esophagojejunostomy after total gastrectomy was performed in 11 consecutive patients diagnosed with early gastric cancer, and their perioperative outcomes were analyzed.

**Results:**

All the operations were successful without conversion to open or laparoscopic surgery and postoperative complications. The total number of patients was 11 (7 males and 4 females). The mean age of the patients was 63.36 ± 10.56 years old. Seven patients were diagnosed with cardia cancer, 3 patients were diagnosed with gastric body cancer, and 1 patient was diagnosed with gastric antrum cancer. The patients’ mean proximal resection margin was 3.18 ± 1.17 cm, the distal resection margin was 6.18 ± 1.40 cm, the mean length of the incision was 4.55 ± 0.69 cm, the mean operative time was 287.27 ± 30.69 min, the mean day of first flatus was 3.27 ± 0.79 days, the mean day of the start of diet was 2.91 ± 0.94 days, the mean postoperative hospital stay was 11.45 ± 5.13 days, and the mean operative blood loss was 47.27 ± 31.33 ml. No complications were observed during anastomosis, and the median anastomosis time was 19.5 min. The mean number of lymph node dissections was 17.91 ± 4.59, the mean number of positive lymph nodes was 0.45 ± 0.69, all patients were diagnosed with stage I–II gastric cancer, and the mean maximum diameter of the tumor was 2.67 ± 1.30 cm. All the patients had a smooth hospital discharge.

**Conclusion:**

A novel robotic gastrectomy with intracorporeal π-shaped EJS for esophagojejunal anastomosis described and shows acceptable resulted. This technique has the potential to offer better short-term surgical outcomes and overcomes the drawbacks of laparoscopy with a decreased risk of complications during and after surgery.

## Introduction

Universally, gastric cancer is the third most common cause of mortality and is the fourth most commonly diagnosed cancer [1]. Gastric cancer is usually diagnosed with metastasis in the advanced stage. There are reports that depending on the diagnosis, the overall survival ranges from 5 to 90% [2].

In China, gastric cancer is the third leading cause of cancer-related death, and every year, it effects roughly 400,000 patients. Surgical resection is still the primary option, despite the advances in combination therapy [3].

Laparoscopic gastrectomy for gastric cancer was initially introduced by Kitano et al. in 1993 [4]. After that, the procedure became more popular, and it is now one of the standard procedures (minimally invasive) for the treatment of early gastric cancer (EGC). In Korea, from 2004 to 2009, the number of laparoscopic surgeries for gastric cancer increased from 740 to 3783 [5]. Due to advances in surgical instruments, surgical experience and a lot of clinical evidence regarding laparoscopic gastrectomy, which was collected over last two decades, especially from Korea and Japan, some experts have extended the use of minimally invasive laparoscopic gastrectomy for gastric cancer from early gastric cancer (EGC) to advanced gastric cancer (AGC), because it is associated with a short hospital stay, earlier bowl moment return, a short bed stay, a decreased inflammatory response, less postoperative pain, and equivalent oncological aspects compared to open surgery [6].

Indeed, a recent feasibility study of laparoscopic total gastrectomy (LTG) for clinical stage I gastric cancer, as a prospective multi-center phase II clinical trial (KLASS 03), revealed that the postoperative morbidity and mortality rates were 20.6 and 0.6%, respectively, the morbidity incidence after LTG was not significantly different from that reported previously for open total gastrectomy (OTG). This finding indicated that when LTG was performed by experienced surgeons, the postoperative morbidity and mortality for patients with clinical stage I gastric cancer was acceptable [7]. However, in case of advanced gastric cancer (AGC), there is a risk of serious postoperative complications due to the required extensive lymphadenectomy and the adjacent organ resection [8]. Furthermore, in spite of the advantages and rapid adoption of laparoscopic gastrectomy, there are still technical issues, with regard to the type of anastomosis, lymphadenectomy, and the oncological safety, in advanced gastric cancer [9, 10]. There is an increase in the number of surgeons performing laparoscopic total gastrectomy. However, the frequency is constant due to reconstruction technical difficulties and complications, such as strictures or anastomotic leakage. Thus, the rate of postoperative complications is higher in laparoscopic-assisted total gastrectomy at the site of the esophagojejunostomy [11]. For gastric cancer, LG has been rapidly adopted in Japan and Korea. At first, EGC with an acceptable limited D1 lymphadenectomy was preferred. However, now, the standard in Japan and Europe is extended to total gastrectomy (TG), with D2 extensive lymph node dissection [12, 13]. The regional lymph node stations of the stomach are divided into three groups by The Japanese Research Society for Gastric Cancer (JRSGC).

To overcome technical limitations of laparoscopic surgery, such as 2D vision, instrument movement limitations, and the unnatural position of the surgeon, robotic surgery was introduced [14, 15]. The robotic system makes it easy for surgeons to perform complex surgical procedures, such as total gastrectomy, lymphadenectomy, and intracorporeal anastomosis, as it allows freedom of movement and a clear 3D view of the surgical field [16]. Studies show that robotic-assisted gastrectomy is more efficient and feasible than laparoscopic-assisted gastrectomy [17, 18]. It is reported that the use of a robotic system overcomes the shortcomings of laparoscopy in gastrointestinal surgery [19]. It is also reported that robotic gastrectomy is associated with a shorter hospital stay and less blood loss during surgery then laparoscopic-assisted gastrectomy [20, 21]. Normally, robot-assisted total gastrectomy (RATG) takes longer than laparoscopic total gastrectomy (LTG), but the learning curve for RATG is shorter [22–25]. However, with practice and more experience, the surgeon is able to overcome the longer duration issue.

In laparoscopic total gastrectomy, one of the big challenges is the esophagojejunostomy (EJS) [9, 26]. With the advancement of lymphadenectomy techniques and reconstructive procedures, the number of reports on LATG has increased. However, EJS through minilaparotomy is still difficult. To overcome these technical difficulties, new esophagojejunostomy techniques have been devised for TLTG and various methods have been applied for intracorporeal EJS anastomosis.

We used the Da Vinci robotic system to perform a 3-in-1 procedure for esophagojejunostomy using a linear stapler during total gastrectomy, known as a *π*-shaped esophagojejunostomy, which was recently introduced [27]. This was proven to overcome the drawbacks of total laparoscopic GI surgeries in contrast to the previous method, for which the reconstruction of the gastrointestinal tract required a 10–15-cm long incision in the epigastric region [28–30].

## Patients and methods

The intracorporeal *π*-shaped EJS, using a linear stapler, for laparoscopic total gastrectomy (LTG) was performed in the Department of General Surgery at the First Affiliated Hospital of Anhui Medical University. Eleven patients underwent this procedure between September 2016 and December 2017 after signing a written informed consent. All patients were histologically confirmed. RATG was performed followed by the new *π*-shaped EJS anastomosis technique using a linear stapler [27], and the short-term operative outcomes were recorded. The primary assessments included the reconstruction type, proximal resection margin, distal resection margin, number of lymph nodes retrieved, estimated blood loss, operative complications, anastomosis time, surgery duration, postoperative first flatus, postoperative hospital stay, and the mortality and morbidity during the first 60 days after the surgery. The feasibility and safety of the procedure were also evaluated. All the procedures were performed with the Da Vinci robotic surgical system.

All the operations in this study were performed by one surgeon (Professor Yongxiang Li) who has performed more than 1000 laparoscopic-assisted or totally laparoscopic gastrectomies since 2008 and 25 robotic-assisted or totally robotic gastrectomies since 2016.

## Indications

The indications for robotic-assisted gastrectomy for gastric cancer were initially similar to the laparoscopic-assisted surgery and were based on the recommendations of the Japanese treatment guideline for gastric cancer, which was clinically diagnosed early gastric cancer, with no evidence of lymph node metastasis. In some cases, robotic-assisted total gastrectomy (RATG) was performed in serosa negative gastric cancer with no lymph node metastasis and in T1 stage with perigastric lymph node involvement [31, 32]. In the serosa involved cancers, neither laparoscopic- nor robotic-assisted gastrectomy are indicated due to the risk of peritoneal seeding metastasis [33], and the use of minimally invasive surgery for advanced gastric cancer is still controversial [34]. Other limitations for minimally invasive surgery are the tumor size and the requirement of a multi-visceral resection [33]. The number and extend of lymph nodes retrieved was in accordance with the Japanese classification of gastric carcinoma guidelines [35].

## Procedure

**Fig. 1:**
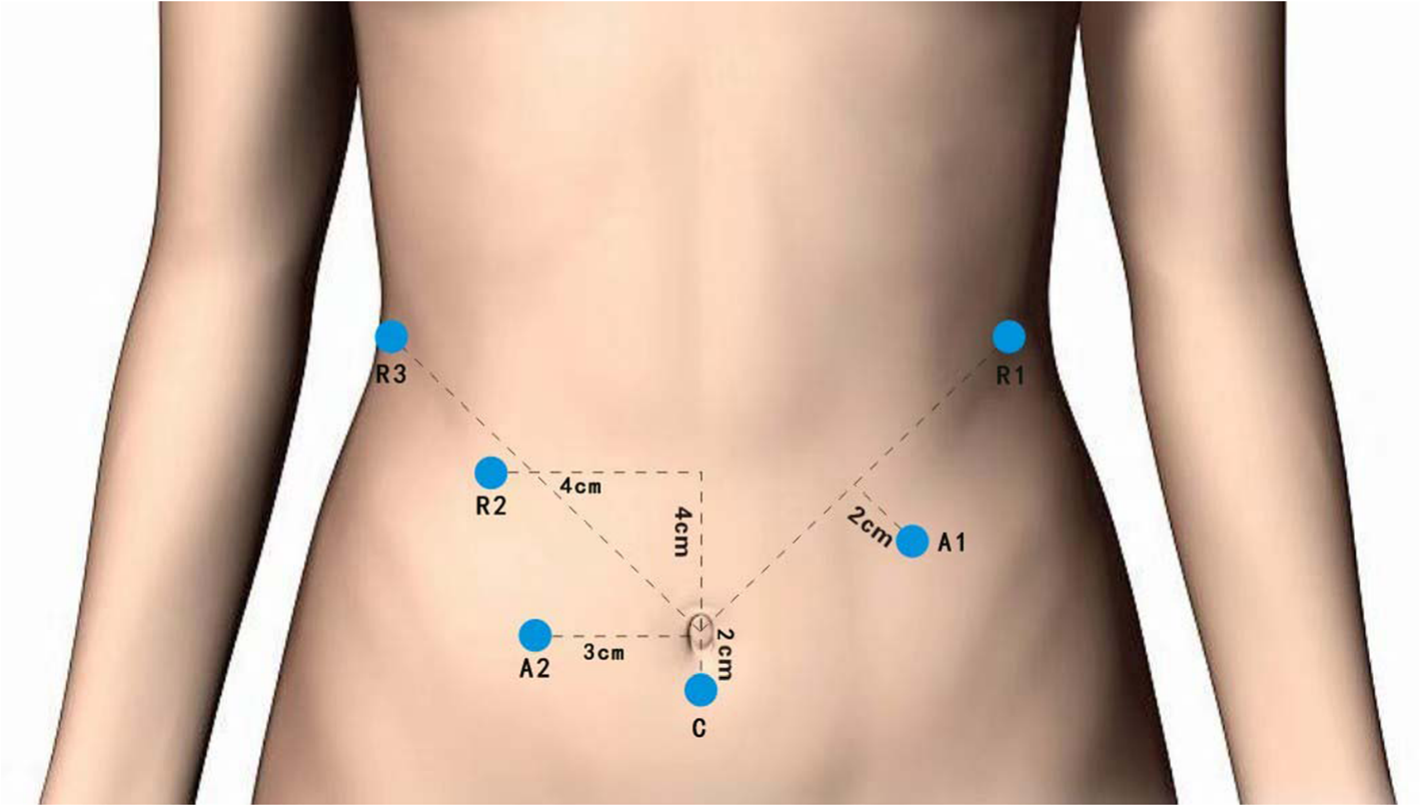
Port placement. A1, A2 two surgical assistant ports; C camera port; and 1, 2, and 3 ports for the robotic arms

The patients were placed in the reverse Trendelenburg position, with 30° elevated legs on a split table. After the administration of general anesthesia, the abdominal cavity was punctured using a veress needle under the umbilicus, and carbon dioxide gas was used to establish the pneumoperitoneum, with an abdominal pressure of 13–15 mmHg, keeping the gas flow at 20 L/min-40 L/min. The first 12-mm trocar for the 3D (three-dimensional) laparoscopic camera was inserted 2 cm below the umbilicus. Next, two 12-mm trocars, for the surgical assistant and three 8-mm trocars, for the robotic arms, were inserted using the laparoscopic camera for visualization. The trocar positions are shown in Fig. 1.

**Fig. 2:**
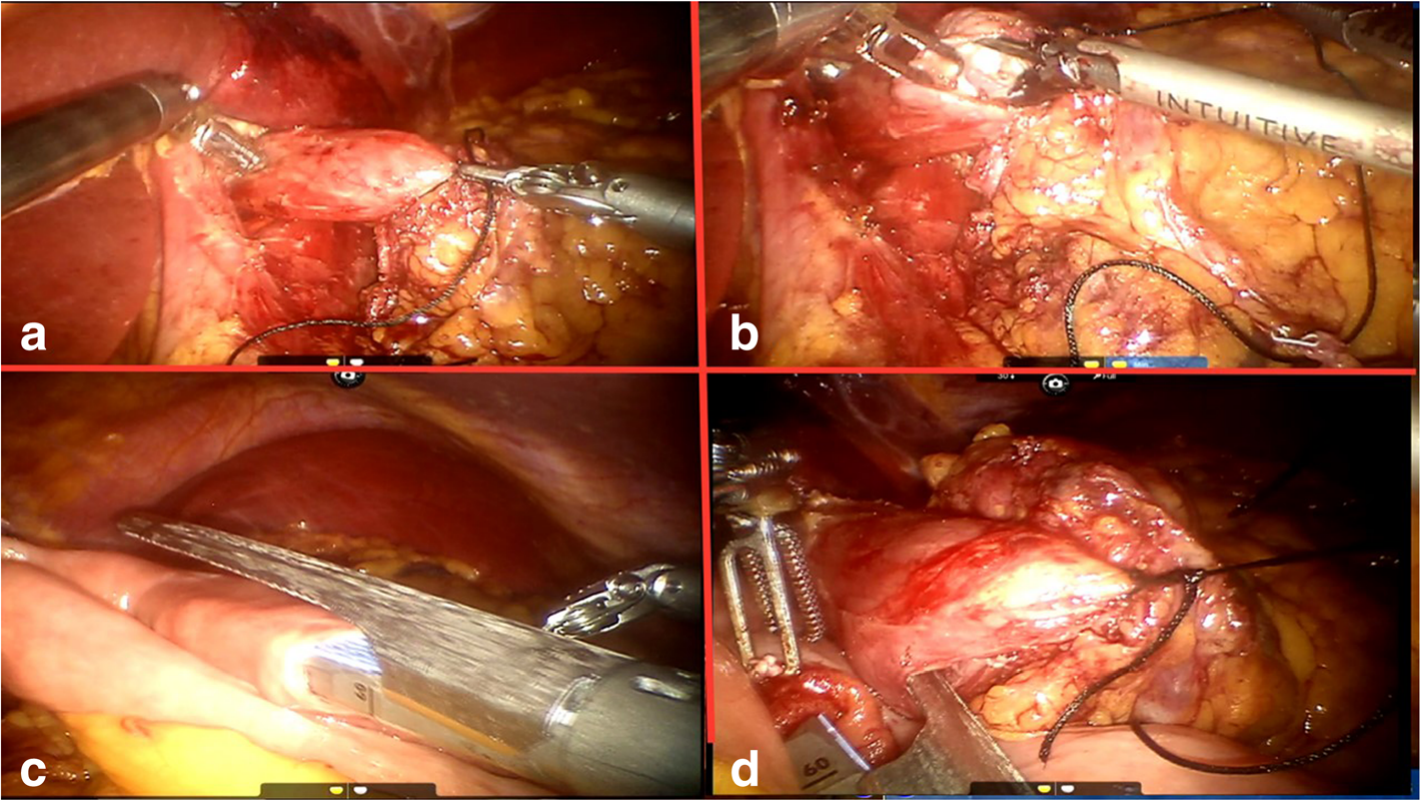
**a** Ligation and retraction of the esophagus. **b** Making an incision on the right side of the esophagus. **c** Placing one prong of the stapler in the jejunal enterotomy. **d** Bringing the esophagus and jejunum in contact

After the complete retroperitoneal detachment of the stomach and lymph node dissection in accordance with the Japanese classification of gastric carcinoma guidelines [34], the abdominal esophagus was separated from the surrounding tissue around the esophagogastric junction and was fully mobilized. The pylorus has been fully detached, and the ECR 60 W (Ethicon Echelon Flex 60 mm Articulating Stapler White) linear stapler was used by the assistant to transect duodenum was transected 2 cm distal to the pylorus. To facilitate the downward traction of the esophagus and to prevent the gastric content spillage, the esophagus was ligated by a belt (Fig. 2a). For the insertion of the linear stapler, while retracting the abdominal esophagus, an incision was made on the right side of the distal esophagus 2–3 cm above the esophagogastric junction or the tumor location (Fig. 2b), and another small enterotomy was made 20–30-cm distal to the ligament of Treitz on the anti-mesenteric side of the jejunum. The first assistant port was used to insert the 60-mm linear stapler. One prong of the stapler was placed in the jejunal enterotomy from the proximal side (Fig. 2c). The esophagus was then retracted, and the other prong of the stapler was inserted in the incision that was made on the right side of the distal esophagus. The stapler was then maneuvered to align the jejunum and esophagus in an antecolic fashion to bring them in contact (Fig. 2d).

**Fig. 3:**
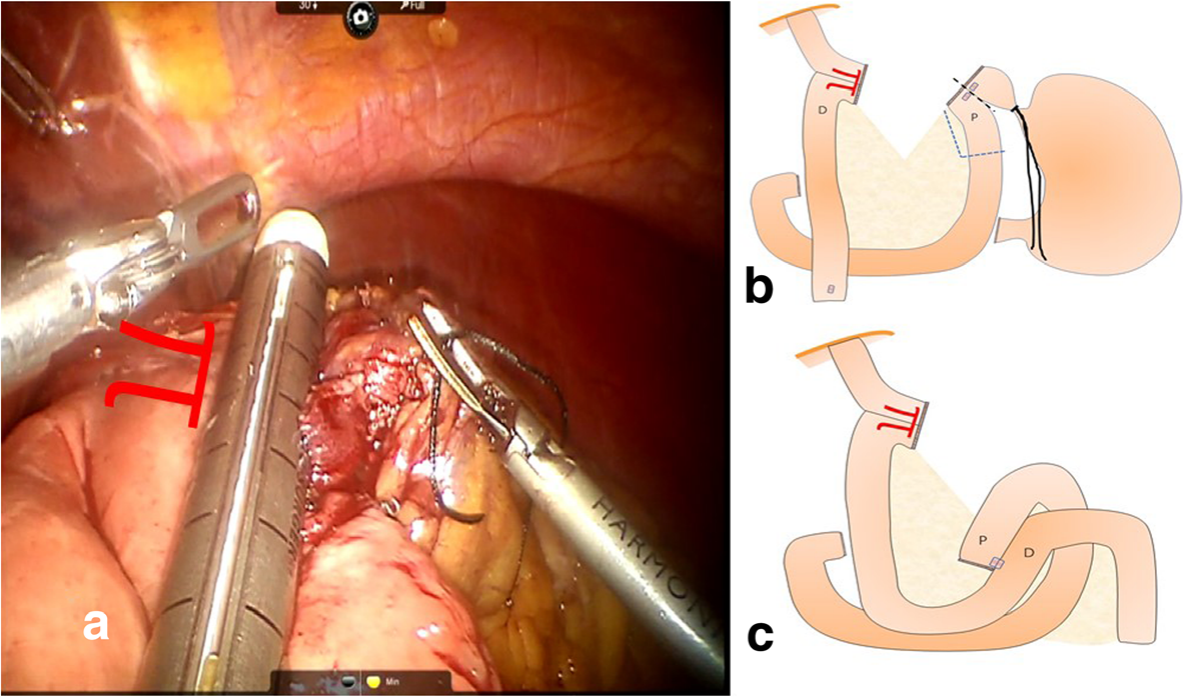
**a** Converting the two holes into single entry hole to establish functional end-to-end esophagojejunostomy by firing the stapler. **b** Stomach was removed only along the black line or with a small segment of the proximal jejunum along blue line. **c** Intracarporeal side-to-side jejunojejunostomy was performed (D distal, P proximal)

The stapler was fired, converting the two holes into single entry hole to establish a functional end-to-end esophagojejunostomy. In addition to the common entry hole from the previous procedure, another small hole was made in the jejunal mesentery. A 60-mm stapler was inserted via the first or second assistant port in the hole made in the jejunal mesentery to create a complete common entry hole, and the sides were checked to make sure the common entry hole was completely severed (Fig. 3a).

The closure of the common entry hole and the division of the esophagus and the jejunal division were performed in a single stapling. In some cases, additional stapling or clipping was performed to complete the closure and division, resulting in the accomplishment of the *π*-shaped intracorporeal esophagojejunostomy. Reinforcement sutures were made at the esophagojejunal junction site and stump. The stomach was removed from the jejunum along the black line. When it was difficult to divide the jejunal mesentery or reduced the tension at the esophagojejunostomy site using the entry hole, a small segment of the proximal jejunum could be removed with the stomach along the blue line (Fig. 3b). Then, intracarporeal side-to-side jejunojejunostomy was performed between the proximal jejunal limb and part of the jejunum 50 cm distal to esophagojejunal anastomosis using a 60-mm linear stapler (Fig. 3c). And then the closure of the common entry hole was made by a 45-mm stapler or suture.

## Results

This study included 11 patients (7 males and 4 females), with a mean age of 63.36 ± 10.56 years old. Seven patients were diagnosed with cardia cancer, 3 patients were diagnosed with gastric body cancer, and 1 patient was diagnosed with gastric antrum cancer. The clinic-pathological and surgical results for the all of the patients are shown in Table 1.

**Table 1:** Clinico-pathological and surgical results of all patients

Variable		Values (*N* = 11)
Age (years)		63.36 ± 10.56
Sex	Male	7
	Female	4
Tumor location	Cardia	7
	Gastric body	3
	Gastric antrum	1
Resection margin (cm)	Proximal	3.18 ± 1.17
	Distal	6.18 ± 1.40
Tumor stage	IB	3
	IIA	3
	IIB	5
Mean length of the incision (cm)		4.55 ± 0.69
Operation time (min)		287.27 ± 30.69
Day of first flatus (days)		3.27 ± 0.79
Resumption of liquid diet (days)		2.91 ± 0.94
Postoperative hospital stay (days)		11.45 ± 5.13
Anastomosis time (min)		19.5
Estimated blood loss (mL)		47.27 ± 31.33
No. of retrieved lymph nodes		17.91 ± 4.59
Lymph node metastasis	N0	8
	N1	2
	N2	1
Tumor size (cm)		2.67 ± 1.30
Esophagojejunostomy-related complication (severe- Clavien-Dind ≥ 3)		0
Other complication (Clavien-Dind < 3)	Fever and incision infection	1
	Intestinal paralysis	1
	Reflux symptom	1

Continuous variables are presented as the mean values ± SD

All the operations were successful without conversion to open or laparoscopic surgery. No complications occurred during anastomosis. In addition, there were no severe complications (using Clavien-Dind ≥ 3). Postoperative complications were observed in 3 patients (27%), and these included 1 patient who had a fever and wound infection, 1 patient with intestinal paralysis after surgery, and 1 patient had a reflux symptom (Clavien-Dind < 3). However, the patients’ complications improved during hospitalization after drug treatment and they were discharged smoothly. Importantly, thus far, none of the patients died, and in the follow-up examinations, none of the patients showed abnormal findings. The follow-up examination included plain and enhanced CT scans to assess tumor recurrence. The mean follow-up period was 10.9 months (range 6–22 months).

Since 2008, our center has performed more than 1000 laparoscopic gastric cancer operations. At the present time, the average length of hospital stay after the operation is 12 ± 4.3 days. The average length of the hospital stay after the robotic surgery presented here was 11.45 ± 5.13 days.

## Discussion

Here, we observed satisfying early postoperative outcomes after a Da Vinci robotic gastrostomy with a π-shaped EJS. We performed the anastomosis inrtracorporeally, because studies show the potential benefits of intracorporeal anastomosis over extracorporeal anastomosis [36]. After considering the technical difficulties and the longer operation time needed for intracorporeal anastomosis using laparoscopy, we decided to use the Da Vinci robotic system.

Surgical resection remains the primary and most effective therapy for gastric cancer. Although most gastric cancer patients are currently treated by laparoscopic surgery, multiple reports suggest that the oncological efficiency of laparoscopic-assisted and open gastrectomy is similar. Laparoscopy facilitates microinvasion, which results in a short hospital stay, less stress, a decreased blood loss, and early bowel function return [37, 38]. However, the use of laparoscopy for D2 total gastrectomy is still debated due to difficulties in lymph node dissection, difficulties in intracorporeal digestive tract restoration, and a high risk of anastomosis leakage [10, 11, 39]. In the past, laparoscopic gastrectomy was referred to as laparoscopic-assisted gastrectomy (LAG), because a mini laparotomy was needed for the extracorporeal reconstruction and specimen extraction, which caused traumatic stress to the patients. The widespread utilization of minimally invasive surgery for gastric cancer is circumscribed by the intricacy of performing an extended D2 lymphadenectomy. Utilizing robot-assisted surgery facilitates this surgical step.

The first robot-assisted gastrectomy was reported by Giulianotti et al. in 2003 [40]. The flexible free movement of the instruments, the precision, the physical tremor elimination, the stability, and the 3D visualization of the robotic system help surgeons to overcome the weaknesses of conventional laparoscopic-assisted surgery. The indications for robotic and laparoscopic surgery were initially same, and both are preferred for early gastric cancer. However, robotic gastrectomy retrieves more lymph nodes, to the same extent as lymphadenectomy, especially in the extraperigastric area [41]. The freedom of movement and better imaging enables surgeons to retrieve a significant number of splenic hilar or suprapancreatic lymph nodes, which is crucial for D2 lymph node dissection. Compared to laparoscopic surgery, there is no obvious difference in the postoperative hospital stay, because the operative time for robotic surgery is longer and the number of trocars used is same as laparoscopic surgery. The longer duration of the robotic surgery is often debated, but the safety and efficiency of the surgery and the shorter learning curve always attract surgeons. Comparative studies show that the robotic learning curve is shorter than that of the laparoscopic method [42]. The use of a robotic system significantly decreases the intraoperative blood loss compared to open and laparoscopic surgeries [25]. The decreased operative blood loss is believed to have potential oncological benefits, because there is a reduction in the dissemination of cancer cells and the postoperative immune function [43–45].

Digestive tract reconstruction is the most important step after gastrectomy. In LAG, a small incision in the epigastric region of the abdomen is necessary for gastrointestinal tract reconstruction anastomosis because of the visualization difficulties and movement limitations. The study conducted by Woo et al. [25] surprisingly showed that the postoperative hospital stay of the robotic gastrectomy patients was longer than that of laparoscopic-assisted patients. Later, Wall et al. [46] noted that the benefits of robotic surgery were limited by the extracorporeal digestive tract restoration. Thus, surgeons began to consider and develop an intracorporeal reconstruction technique to improve the postoperative and intraoperative outcomes. Hur et al. [47] successfully achieved and published anastomosis (esophagojejunostomy, gastroduodenostomy, and gastrojejunostomy), for the first time, using a robot-sewing technique performed within the abdominal cavity. Since then, many attempts have been made to develop new techniques that are effective, feasible, and economic.

The aim of this report was to describe a robotic *π*-shaped EJS technique and its short-term results in totallyrobotic gastrectomies with D2-lymphadenectomy for gastric cancer, utilizing the Da Vinci Surgical System. In this report, we demonstrated the feasibility of the robotic system in gastrectomy for gastric cancer with intracorporeal *π*-shaped esophagojejunostomy and its efficiency in gastrointestinal tract reconstruction.

## Conclusion

The novel robotic gastrectomy with intracorporeal *π*-shaped esophagojejunostomy procedure for esophagojejunal anastomosis presented here is feasible, effective, and safe. This report shows that robotic *π*-shaped esophagojejunostomy can be applied safely and effectively in patients with gastric cancer needing total gastrectomy. This technique may provide better surgical outcomes and overcome the drawbacks of laparoscopy, with a decreased risk of complications during and after surgery. The presentation of this novel technique lays the foundation for establishing a randomized controlled study with a larger sample size and a longer follow-up period.

## Abbreviations

AGC: Advanced gastric cancer
EGC: Early gastric cancer
EJS: Esophagojejunostomy
GI: Gastrointestinal
LAG: Laparoscopic-assisted gastrectomy
LATG: Laparoscopy-assisted total gastrectomy
LG: Laparoscopic gastrectomy
RATG: Robotic-assisted total gastrectomy
TG: Total gastrectomy
TLTG: Totally laparoscopic total gastrectomy
TRTG: Totally robotic total gastrectomy
